# Spatial transcriptomic profiling reveals fibroblast and inflammatory signatures in calcinosis of chronic cutaneous lupus

**DOI:** 10.1016/j.xjidi.2026.100504

**Published:** 2026-06-25

**Authors:** Aaron Bao, Saloni Patel, Sewon Kang, Jaroslaw Jedrych, Martin Alphonse, Jun Kang

**Affiliations:** 1 Department of Dermatology, Johns Hopkins University School of Medicine, Baltimore, Maryland, USA

**Keywords:** Autoimmune, Calcinosis cutis, Cutaneous lupus, Lupus erythematosus

## Abstract

Calcinosis cutis represents a debilitating complication of chronic cutaneous lupus erythematosus; however, its underlying molecular mechanisms remain poorly understood. We performed spatial transcriptomic profiling on skin biopsies from 4 patients with chronic cutaneous lupus erythematosus: 2 with calcinosis cutis and 2 without. Analysis of 87,730 cells across 6 major cell types revealed distinct molecular signatures associated with calcification. Fibroblasts in calcinosis cutis lesions exhibited significant upregulation of osteogenic and extracellular matrix genes, including periostin, collagen family members, and matrix metalloproteinase 2, accompanied by enrichment of pathways related to extracellular matrix organization and ossification. Endothelial cells demonstrated activation of hypoxia-responsive pathways, particularly hypoxia-inducible factor 2-alpha. Macrophages showed elevated chemokine receptor expression with corresponding ligand upregulation in endothelial cells, establishing a potential chemotactic recruitment axis. Spatial analysis revealed that these pathological features were concentrated in pericalcinosis regions, with reduced intercellular distances between functionally related cell populations. These findings demonstrate coordinated fibroblast osteogenic reprogramming, endothelial hypoxic responses, and immune cell activation within organized pericalcinosis niches. This spatial transcriptomic characterization of lupus-associated calcinosis cutis identifies potential therapeutic targets and reveals mechanisms resembling those in other calcifying disorders.

## Introduction

Dystrophic calcinosis cutis (CC), a debilitating complication of rheumatic skin disease, is characterized by the pathological deposition of calcium salts within dermal and subcutaneous tissues (Le and Bedocs, 2023). This ectopic mineralization process occurs through dystrophic calcification in the setting of normal serum calcium and phosphate levels, distinguished from metastatic calcification, which is associated with metabolic disorders.

The prevalence of CC varies significantly across autoimmune connective tissue diseases. Systemic sclerosis demonstrates the highest incidence, affecting 25–40% of patients within the first decade of disease (Valenzuela et al, 2022), whereas dermatomyositis shows involvement in up to 20% of adult patients (Davuluri et al, 2022). In contrast, CC remains a relatively uncommon manifestation in cutaneous lupus erythematosus (CLE), with forms of chronic CLE (CCLE), such as lupus panniculitis/profundus and discoid lupus erythematosus, thought to carry a higher risk (Achebe et al, 2020; Korekawa et al, 2015).

The clinical spectrum of CC ranges from asymptomatic subcutaneous nodules to extensive tumoral calcifications that significantly impact patient’s QOL. Lesions demonstrate an anatomical predilection for areas of mechanical stress and previous tissue injury, including the upper extremities, buttocks, and periauricular regions. In rheumatic skin diseases, calcium deposits tend to localize to sites of prior cutaneous inflammation, suggesting a mechanistic link between chronic lupus-associated tissue damage and subsequent mineralization (Balin et al, 2012). Complications include ulceration with secondary bacterial infection, mechanical joint restriction, and chronic pain syndromes.

Despite the substantial clinical burden imposed by CC, fundamental knowledge gaps persist regarding the cellular and molecular mechanisms driving this complication. To date, no transcriptomic studies have specifically investigated CC in cutaneous lupus. Although a recent spatial transcriptomic study of calcinosis in dermatomyositis identified macrophage-driven inflammation, matrix metalloproteinase dysregulation, and osteopontin upregulation as key disease-associated pathways (Parks et al, 2025), the molecular landscape of calcinosis in CCLE remains uncharacterized.

Recent technological advances in spatial transcriptomics offer novel opportunities to dissect tissue-level pathology while preserving critical spatial relationships between cell populations (Chen et al, 2023; Luecken and Theis, 2019). This approach has already transformed our understanding of cutaneous lupus pathogenesis, revealing type I IFN–dominant microenvironments, aberrant fibroblast activation in discoid lupus erythematosus, and endothelial dysfunction (Sarkar et al, 2018; Tydén et al, 2017; Wenzel et al, 2005).

This study leverages spatial transcriptomic profiling to characterize the cellular microenvironment of CC in CCLE. By comparing transcriptional landscapes of CCLE lesions with CC and without CC, we aimed to identify cell type–specific gene expression programs, intercellular communication networks, and spatial organization patterns that distinguish calcifying from noncalcifying lupus lesions. Informed by existing literature on other forms of extracutaneous pathological calcification and insights from related rheumatic diseases, we hypothesized that CC in CCLE is associated with distinct fibroblast activation states, altered vascular biology, and specific inflammatory signatures that collectively promote tissue mineralization. These findings may illuminate therapeutic targets for preventing or reversing this challenging complication in patients with lupus.

## Results

### Patient characteristics and sample overview

This study included 4 patients with CCLE: 2 with biopsy-confirmed CC and 2 without CC (noCC). Demographic and clinical information, the biopsy site, and the treatment at the time of biopsy are summarized in Table 1.

**Table 1:** Patient Demographics and Clinical Characteristics

Characteristic	CC1	CC2	noCC1	noCC2
Age, y	20s	30s	40s	60s
Race^1^	Hispanic	Black	Hispanic	Black
Sex	F	F	F	F
Disease duration, y	2	12	4	7
Autoimmune comorbidities	None	Lupus nephritis, systemic lupus erythematosus	Systemic lupus erythematosus	None
Clinical features at biopsy	Violaceous, reticulated plaque on right breast with erythema, hypopigmentation, and poikiloderma. Underlying firm nodules on deeper palpation. Hypopigmented macules along shin and neck	Multiple hyperpigmented papules and plaques with areas of nodular calcinosis on chest, legs, arms, dorsal hands, conchal bowls, nose, and malar cheeks Erythema and scale along the fingertips, scaling of toes	Violaceous and erythematous, hyperkeratotic plaques with peripheral hyperpigmentation and scale on sun-exposed areas of the face, frontal scalp, and upper arms. Affected areas of the scalp with associated scarring alopecia Fingertips and toes with deep violaceous color and associated scale	Erythematous plaques with central black area with heaped up borders on the frontal scalp Hyperpigmented patch on the lower lip with surrounding erythema Irregularly bordered hyperpigmented patch on the nose, surrounded by mild hypopigmentation
Biopsy reason	Diagnostic biopsy	Research consented biopsy	Diagnostic biopsy	Research consented biopsy
Biopsy site	Right breast	Left thigh	Right arm	Scalp
Treatment at biopsy	None, treatment naïve	HCQ, MMF, anifrolumab, triamcinolone	None, no treatment one year prior to biopsy	None, no treatment two years prior to biopsy

Abbreviations: CC, calcinosis cutis; F, female; HCQ, hydroxychloroquine; MMF, mycophenolate mofetil; noCC, no calcinosis cutis.

1 Note on race and ethnicity: Patient race and ethnicity categories were retrieved from institutional electronic medical record data recorded during routine clinical care. These variables were collected independently of any funding agency mandate to accurately characterize the clinical and sociodemographic diversity of this cohort.

### Histological characterization and pericalcinosis region definition

**Figure 1:**
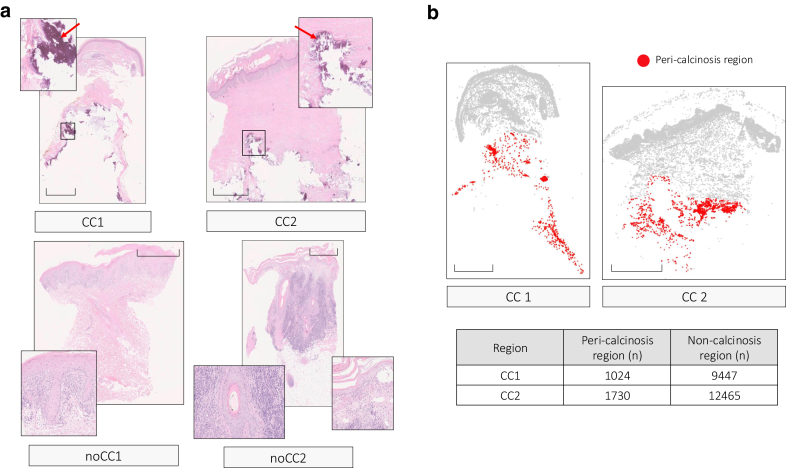
**Histological characterization and pericalcinosis region definition in chronic cutaneous lupus erythematosus samples.** (**a**) Representative H&E-stained sections from 4 patient samples. Calcinosis nodules are denoted with red arrows. Subtle vacuolar interface dermatitis is present in both CC samples. In addition, perifollicular and notable interface dermatitis is seen in noncalcinosis samples. (**b**) Spatial transcriptomic analysis of CC samples showing manually delineated pericalcinosis regions (red) and noncalcinosis regions (gray). Table shows cell counts for each region in CC1 and CC2 samples. Bar = 1 mm. CC, calcinosis cutis; noCC, no calcinosis cutis.

Histological examination revealed distinct architectural patterns between the CC and noCC groups (Figure 1a). CC samples displayed basophilic, irregularly shaped calcium deposits distributed throughout the dermis and subcutaneous tissue, often accompanied by focal tissue disruption with subtle vacuolar interface and perivascular dermatitis patterns. In contrast, noCC samples exhibited classic cutaneous lupus histopathology, including intense interface dermatitis, vacuolar degeneration of the basal layer, and perivascular lymphocytic infiltrates without evidence of mineralization (Table 2).

**Table 2:** Histopathological Features of Study Samples

Sample	Histological Pattern
CC1	Subtle vacuolar interface and perivascular dermatitis with nodular calcium deposits in the dermis
CC2	Subtle vacuolar interface and perivascular dermatitis with nodular calcium deposits in the dermis
noCC1	Intense lichenoid interface dermatitis
noCC2	Intense vacuolar interface, perivascular and periadnexal dermatitis

Abbreviations: CC, calcinosis cutis; noCC, no calcinosis cutis.

For spatial analysis within CC samples, pericalcinosis regions of interest were manually delineated as the tissue areas immediately surrounding visible calcium deposits. These regions constituted 10% (1024 of 10,471 cells) and 12% (1730 of 14,195 cells) of the total analyzed cells in CC1 and CC2 samples, respectively (Figure 1b).

### Spatial transcriptomic profiling and cell-type identification

**Figure 2:**
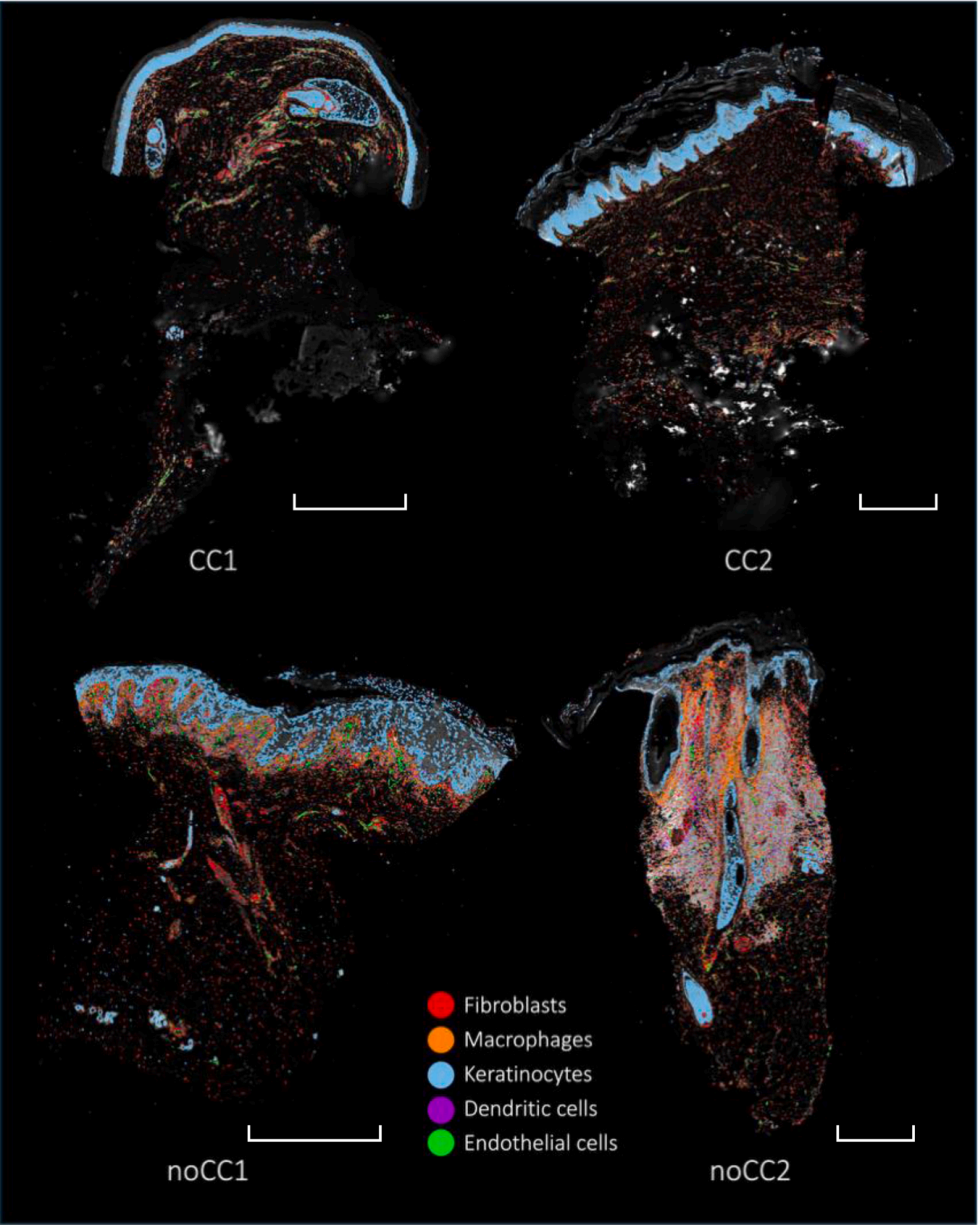
**Spatial distribution of cell types across tissue sections.** Spatial plots showing distribution of 6 identified cell types across all 4 patient samples. Each dot represents a single cell colored by cell type: fibroblasts (red), macrophages (orange), keratinocytes (yellow), dendritic cells (green), endothelial cells (blue), and T cells (purple). Spatial coordinates preserve anatomical relationships. Bar = 1 mm. CC, calcinosis cutis; noCC, no calcinosis cutis.

Spatial transcriptomic analysis successfully captured 87,730 high-quality cells across all tissue sections. Unsupervised clustering identified 6 distinct cell populations: fibroblasts (n = 21,031; 24.0%), keratinocytes (n = 30,718; 35.0%), macrophages (n = 15,351; 17.5%), endothelial cells (n = 4288; 4.9%), dendritic cells (DCs) (n = 2639; 3.0%), and T cells (n = 13,703; 15.6%) (Figure 2).

**Figure 3:**
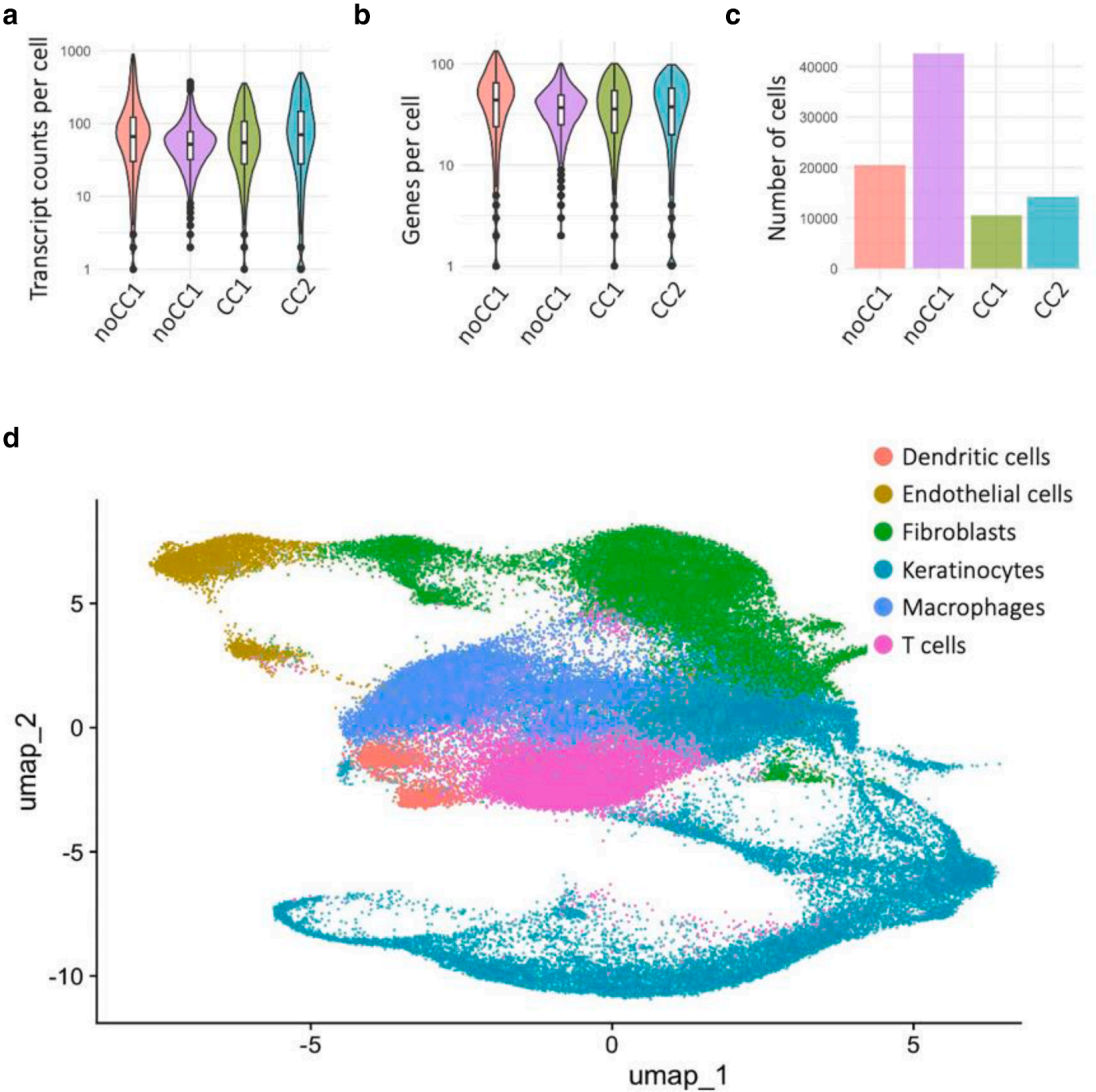
**Quality control metrics for spatial transcriptomic analysis.** (**a**) Violin plots showing distribution of transcript counts per cell across all 4 samples. (**b**) Violin plots showing distribution of genes detected per cell across all 4 samples. (**c**) Bar plot showing total number of high-quality cells captured per sample after quality control filtering. Horizontal dashed line indicates median across samples. (**d**) UMAP projection of 87,730 cells colored by identified cell type. CC, calcinosis cutis; noCC, no calcinosis cutis; UMAP, Uniform Manifold Approximation and Projection.

**Figure 4:**
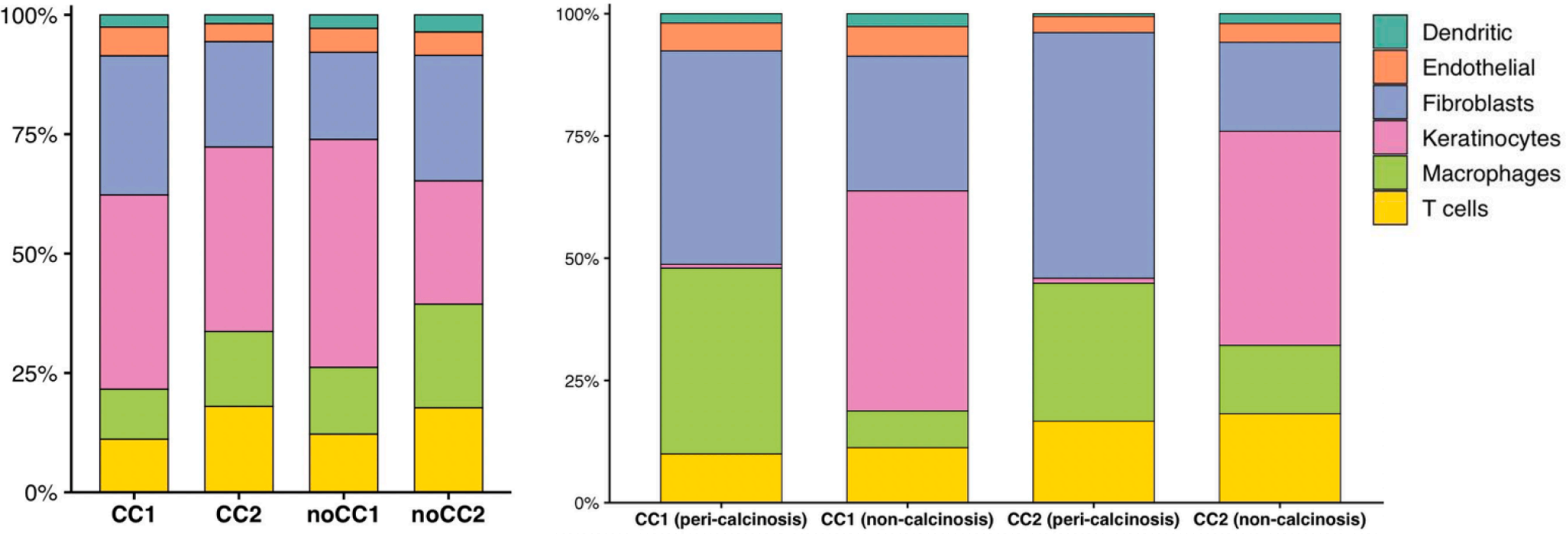
**Visual representation of cell types between samples (left) and calcinosis regions (right)**.

Quality control metrics demonstrated consistent performance across samples, with median transcript detection ranging from 45 to 65 genes per cell and mean transcript counts ranging from 150 to 200 per cell (Figure 3). Cell population proportions were also calculated for each sample (Table 3 and Figure 4).

**Table 3:** Cell Counts and Types for Each Sample

Cell Type	CC1	CC2	noCC1	noCC2
Dendritic	269 (3)	262 (2)	611 (3)	1498 (4)
Endothelial	632 (6)	536 (4)	1076 (5)	2043 (5)
Fibroblasts	3050 (29)	3134 (22)	3916 (19)	10,932 (26)
Keratinocytes	4260 (41)	5481 (39)	10,237 (50)	10,740 (25)
Macrophages	1096 (10)	2228 (16)	2997 (15)	9031 (21)
T cells	1165 (11)	2554 (18)	2616 (13)	7368 (17)

Abbreviations: CC, calcinosis cutis; noCC, no calcinosis cutis.

### Differential gene expression analysis

#### Fibroblasts

**Figure 5:**
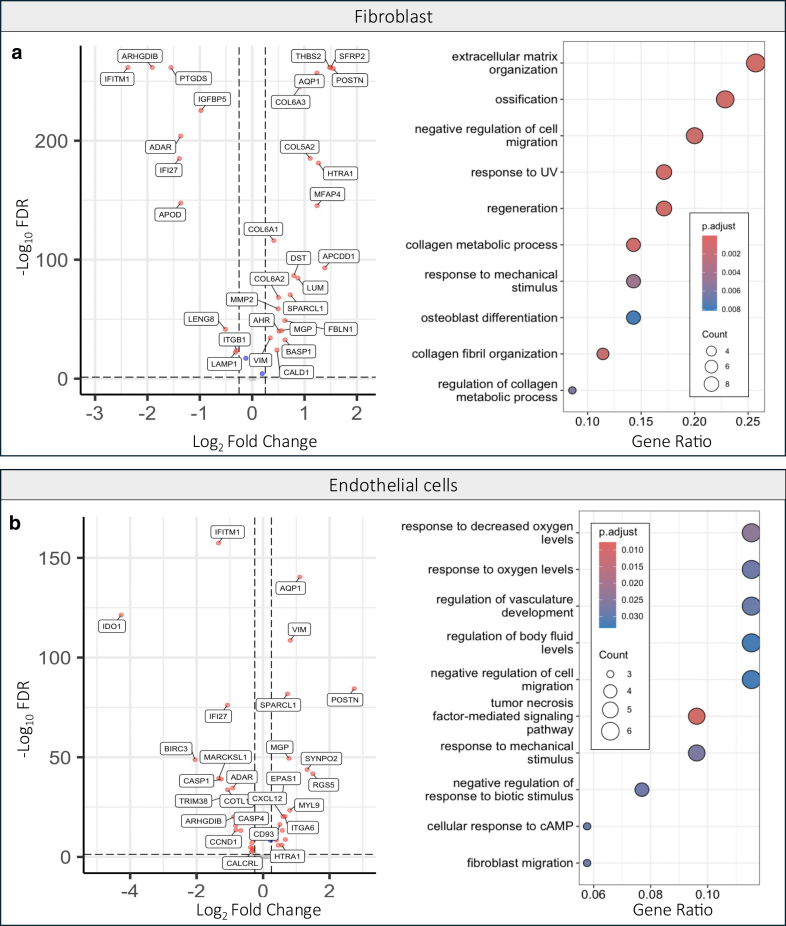
**Differential gene expression in fibroblasts and endothelial cells from CC lesions.** Volcano plots showing differentially expressed genes in (**a**) fibroblasts and (**b**) endothelial cells comparing CC with noCC samples. X-axis represents log_2_ FC; y-axis represents −log_10_ FDR. Red points indicate significantly dysregulated genes (FDR < 0.05, log_2_FC > 0.25). Key genes are labeled. Dot plots show gene ontology enrichment analysis for significantly differentially expressed genes in (**a**) fibroblasts and (**b**) endothelial cells. Gene ratio indicates the proportion of differentially expressed genes in each pathway. Dot size represents gene count; color intensity represents adjusted *P*-value. CC, calcinosis cutis; FC, fold change; FDR, false discovery rate; noCC, no calcinosis cutis.

Fibroblasts in CC samples exhibited extensive transcriptional reprogramming, with 33 genes showing significant differential expression (false discovery rate [FDR] < 0.05, log_2_ fold change [FC] > 0.25). The upregulated signature was dominated by extracellular matrix (ECM) genes, with *POSTN* demonstrating the highest FC (log_2_FC = 1.6, FDR < 0.001). Structural matrix components, including *COL6A1* (log_2_FC = 0.4), *COL6A3* (log_2_FC = 0.9), *COL5A2* (log_2_FC = 1.2), and genes involved in fibrillar organization (*FBLN1*, *LUM*, and *SPARCL1*), were coordinately upregulated. Concurrently, matrix remodeling enzymes, such as *MMP2* (log_2_FC = 0.5) and *HTRA1* (log_2_FC = 1.2), also showed increased expression (FDR < 0.001) (Figure 5a).

Pathway enrichment analysis identified significantly enriched biological processes in CC fibroblasts, with “ECM organization" showing notable enrichment (FDR < 0.001, gene ratio = 0.27). Additional significantly enriched pathways included "ossification" (FDR = 0.003), "response to mechanical stimulus" (FDR = 0.005), and "collagen fibril organization" (FDR = 0.002).

#### Endothelial cells

CC-associated endothelial cells displayed a differential gene expression signature enriched for oxygen-sensing pathways. *EPAS1* showed upregulation (log_2_FC = 0.6), accompanied by downstream targets including *AQP1* (log_2_FC = 1.2) and *RGS5* (log_2_FC = 1.7) (FDR < 0.001). Functional annotation revealed enrichment for hypoxia response pathways: "response to decreased oxygen levels" (FDR = 0.02), "response to oxygen levels" (FDR = 0.03), and "regulation of vasculature development" (FDR = 0.03) (Figure 5b).

#### Immune cells

**Figure 6:**
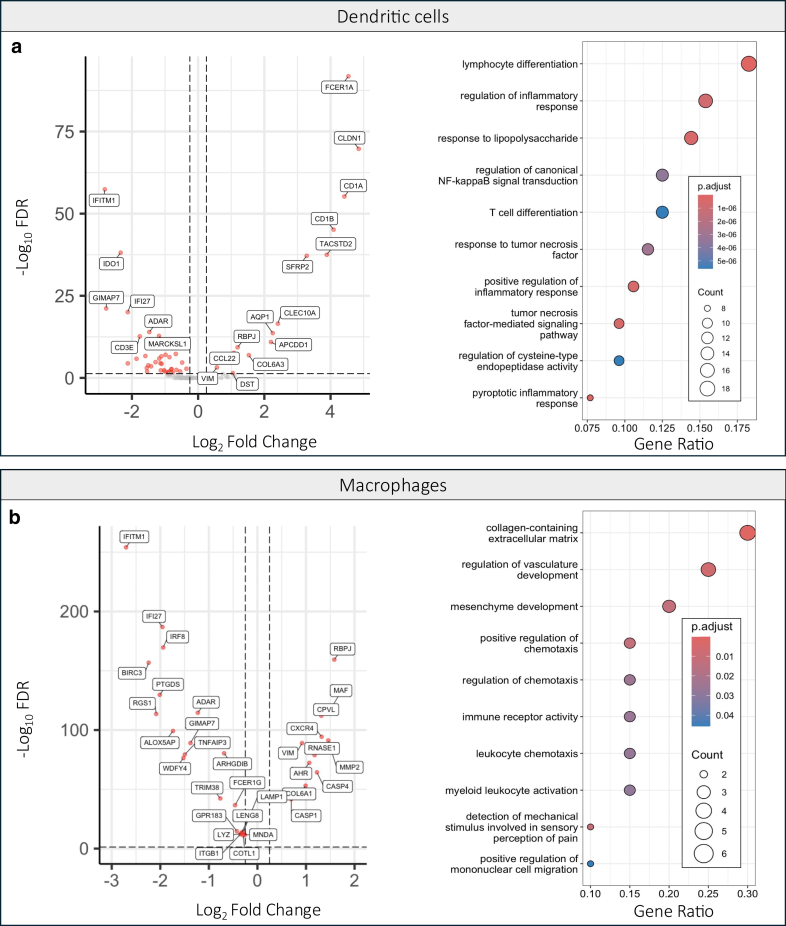
**Differential gene expression in dendritic cells and macrophages from CC lesions.** Volcano plots showing differentially expressed genes in (**a**) dendritic cells and (**b**) macrophages comparing CC with noCC samples. X-axis represents log_2_ FC; y-axis represents −log_10_ FDR. Red points indicate significantly dysregulated genes (FDR < 0.05, log_2_FC > 0.25). Key genes are labeled. Dot plots show gene ontology enrichment analysis for significantly differentially expressed genes in (**a**) dendritic cells and (**b**) macrophages. Gene ratio indicates proportion of differentially expressed genes in each pathway. Dot size represents gene count; color intensity represents adjusted *P*-value. CC, calcinosis cutis; FC, fold change; FDR, false discovery rate; noCC, no calcinosis cutis.

DCs in CC lesions exhibited activation markers, including elevated *FCER1A*, *CD1A*, and *CD1B* expression. Pathway analysis of DC transcripts revealed enrichment for lymphocyte differentiation, regulation of inflammatory response, and NF-κB signaling (all FDR < 0.001) (Figure 6a).

Macrophage populations demonstrated elevated *CXCR4* expression (log_2_FC = 1.3, FDR < 0.001). Additional macrophage-enriched pathways included the regulation of chemotaxis, immune receptor activity, and myeloid cell activation (all FDR < 0.05) (Figure 6b).

In contrast, keratinocyte and T-cell populations showed minimal transcriptional differences between the CC and noCC groups, with fewer than 10 significantly altered genes per cell type.

### Pericalcinosis regional analysis

**Figure 7:**
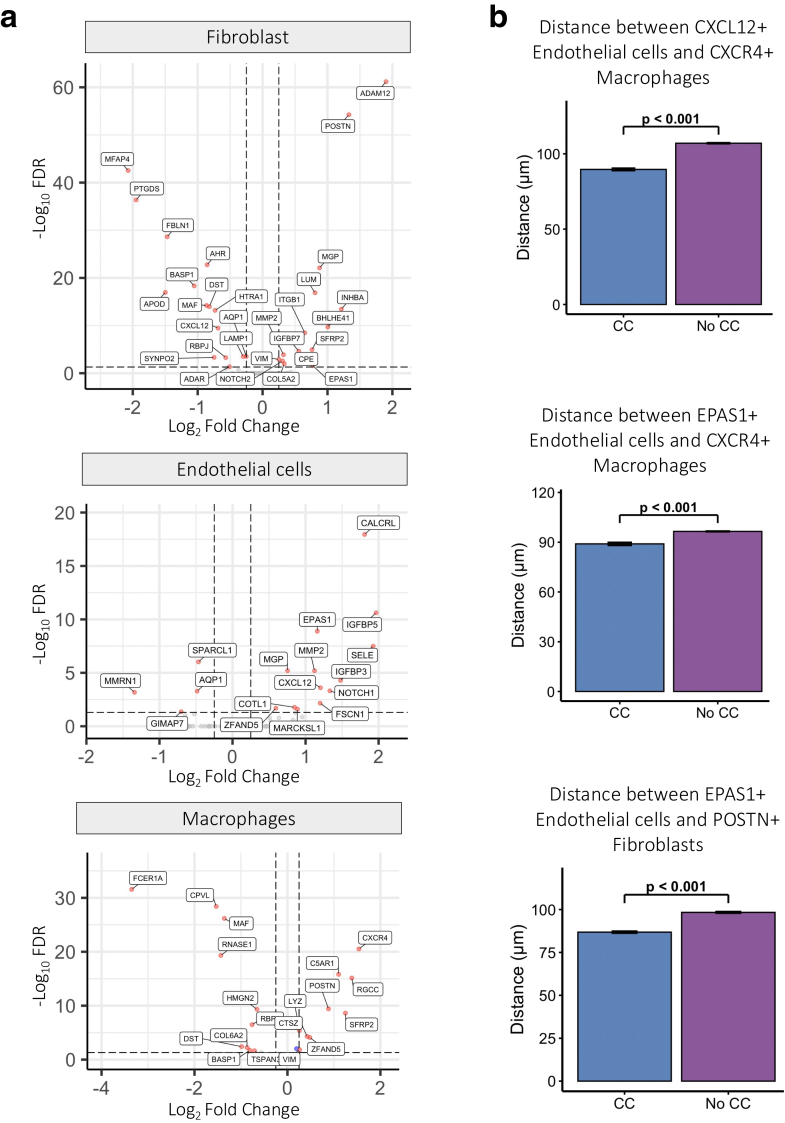
**Spatial analysis of pericalcinosis regions and cellular interactions.** (**a**) Volcano plots showing differentially expressed genes in pericalcinosis versus noncalcinosis regions within CC samples for fibroblasts (top), endothelial cells (middle), and macrophages (bottom). X-axis represents log_2_ FC; y-axis represents −log_10_ FDR. Red points indicate significantly dysregulated genes in pericalcinosis regions (FDR < 0.05, log_2_FC > 0.25). Key genes are labeled. (**b**) Box plots showing intercellular distances between functionally related cell populations in CC versus noCC samples. Box represents interquartile range; horizontal line indicates median; whiskers extend to minimum and maximum values. ∗∗∗*P* < .001 by Wilcoxon rank-sum test. CC, calcinosis cutis; FC, fold change; FDR, false discovery rate; noCC, no calcinosis cutis.

Within CC samples, comparative analysis of pericalcinosis regions versus distant regions revealed distinct region-specific expression patterns across fibroblast, endothelial, and macrophage populations (Figure 7a). Cell population proportions between pericalcinosis and noncalcinosis regions were calculated (Table 4 and Figure 4).

**Table 4:** Cell Counts and Types for Calcinosis Samples Separated by Pericalcinosis and Noncalcinosis Regions

Cell Type	CC1 (Pericalcinosis)	CC1 (Noncalcinosis)	CC2 (Pericalcinosis)	CC2 (Noncalcinosis)
Dendritic	20 (2)	249 (3)	10 (1)	252 (2)
Endothelial	58 (6)	574 (6)	57 (3)	479 (4)
Fibroblasts	447 (44)	2603 (28)	869 (50)	2265 (18)
Keratinocytes	8 (1)	4252 (45)	18 (1)	5463 (44)
Macrophages	389 (38)	707 (7)	488 (28)	1740 (14)
T cells	102 (10)	1063 (11)	288 (17)	2266 (18)

Abbreviations: CC, calcinosis cutis; noCC, no calcinosis cutis.

Among fibroblasts, the most significantly upregulated genes included *ADAM12* and *POSTN*, which also demonstrated the highest FCs in the global CC versus noCC analysis. *SFRP2*, *MGP*, and *LUM* were additionally upregulated in this regional comparison. Endothelial cells showed upregulation of *CALCRL* (log_2_FC = 1.8, FDR < 0.001) and EPAS1 (log_2_FC = 1.2, FDR < 0.001). Notably, *CXCL12* was also significantly upregulated alongside *MMP2*, *SELE*, and *NOTCH1*. Macrophages demonstrated the highest enrichment for *CXCR4* than distant regions, with additional upregulation of *C5AR1*, *POSTN*, *SFRP2*, and *RGCC*. Several genes were concordantly upregulated across both the global CC versus noCC and pericalcinosis regional analyses within their respective cell types, as summarized in Table 5.

**Table 5:** Shared Upregulated Genes Across Global and Pericalcinosis Analyses by Cell Type

Cell Type	Gene List
Fibroblast	*POSTN*, *SFRP2*, *MGP*, *COL5A2*, *MMP2*, *LUM*
Endothelial cells	*EPAS1*, *CXCL12*, *MGP*
Macrophages	*CXCR4*, *SFRP2*

### Spatial cellular interaction analysis

Spatial proximity analysis quantified the intercellular distances between functionally related cell populations. CC samples demonstrated significantly altered spatial organization compared with controls across multiple cell-type pairs.

Specifically, *CXCL12*-expressing endothelial cells showed a reduced mean distance to *CXCR4*-positive macrophages in CC lesions (94.6 μm vs 105.2 μm, *P* < .001). Similarly, *EPAS1*-positive endothelial cells were positioned closer to *CXCR4*-expressing macrophages (89.2 μm vs 98.8 μm, *P* < .001) and POSTN-positive fibroblasts (87.7 μm vs 95.1 μm, *P* < .001) in CC samples (Figure 7b).

**Figure 8:**
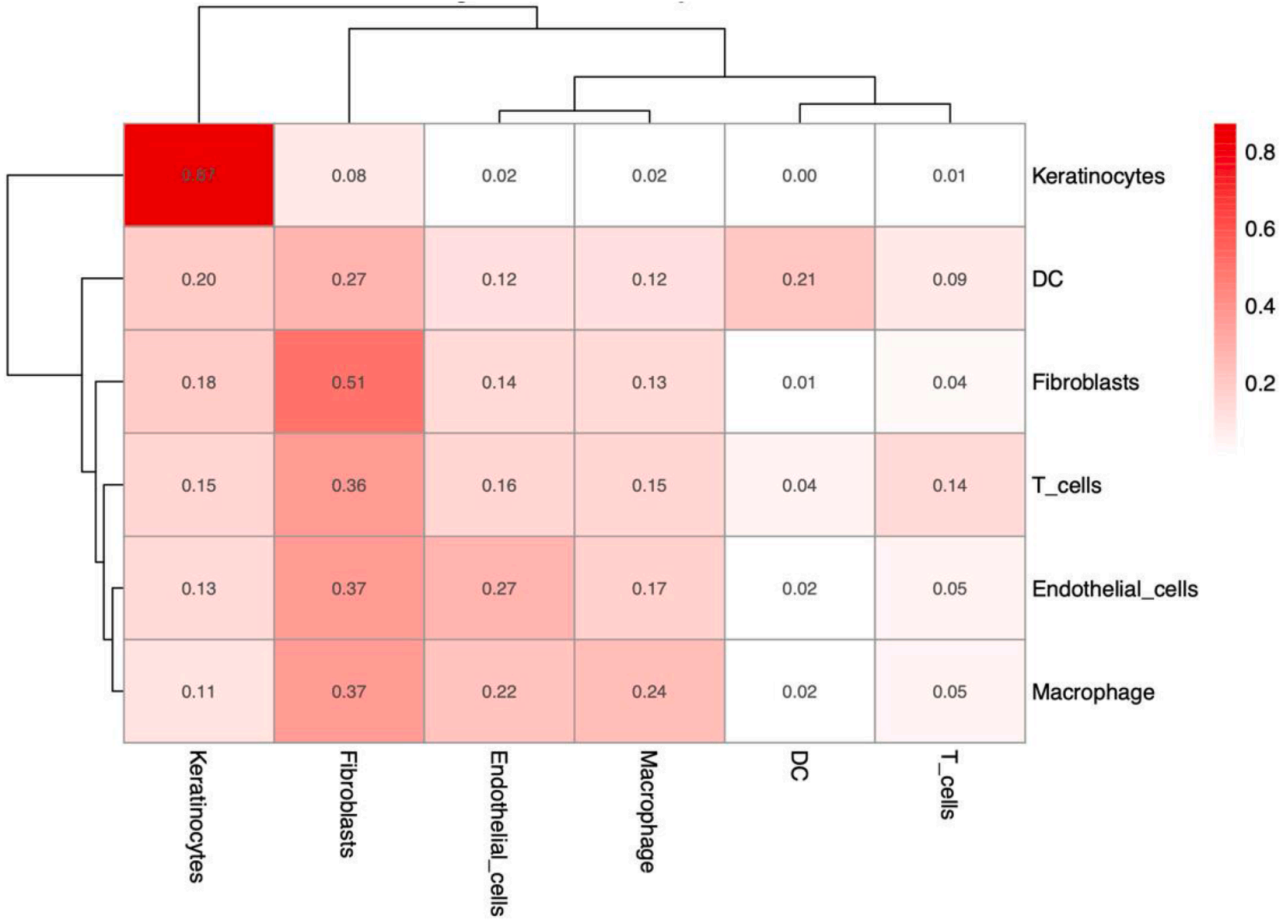
**Neighborhood composition analysis of CC lesions in chronic cutaneous lupus erythematosus.** Heatmap depicting the spatial neighborhood composition of 6 major cell populations identified by spatial transcriptomic profiling in CC samples is shown. Each row represents a cell type of interest; each column represents a neighboring cell type. Values indicate the proportion of neighbors belonging to each cell type within a 50-μm radius, with rows summing to 1. Color intensity reflects neighbor proportion (white, low; red, high). Hierarchical clustering was performed on both rows and columns. CC, calcinosis cutis.

To characterize the cellular organization of CC lesions, we performed spatial neighborhood composition analysis on CC samples, quantifying the proportion of each cell type found within a 50-μm radius of every cell. Fibroblasts emerged as the dominant neighbor across most cell populations, constituting 51% of neighboring cells for other fibroblasts, 37% for macrophages, 37% for endothelial cells, and 36% for T cells. Macrophages and endothelial cells demonstrated reciprocal spatial proximity, with 22% of macrophage neighbors being endothelial cells and 24% being other macrophages. Keratinocytes demonstrated predominant self-clustering, with 87% of their neighboring cells being other keratinocytes. DCs showed the most spatially diffuse neighbor distribution across multiple cell types without a strong preferential association (Figure 8).

### Spatial transcriptomic imaging of key genes and cell populations

**Figure 9:**
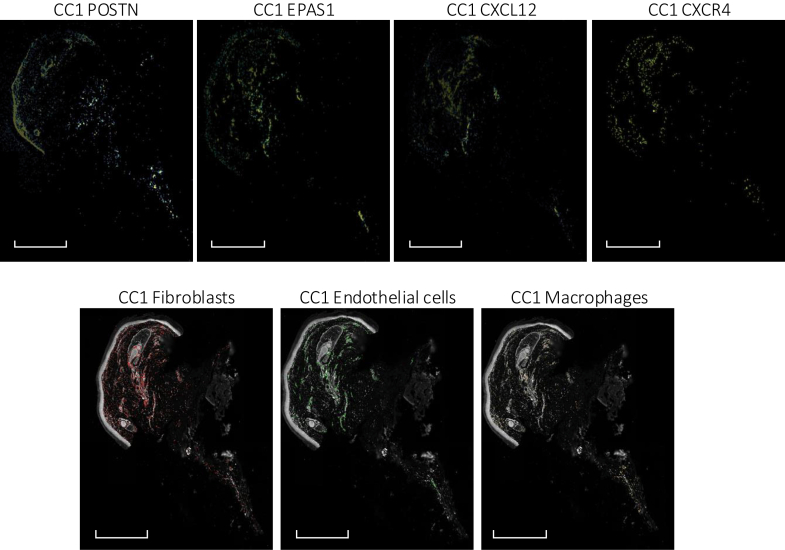
**Spatial representation of genes and cell types in CC1.** Top row: spatial expression maps of key differentially expressed genes in CC1, including POSTN (periostin), EPAS1 (HIF-2α), CXCL12, and CXCR4, visualized using the Xenium In Situ platform. Bottom row: spatial distribution of key cell populations in CC1, including fibroblasts (red), endothelial cells (green), and macrophages (orange). Bar = 1 mm. CC, calcinosis cutis; HIF-2α, hypoxia-inducible factor 2α.

**Figure 10:**
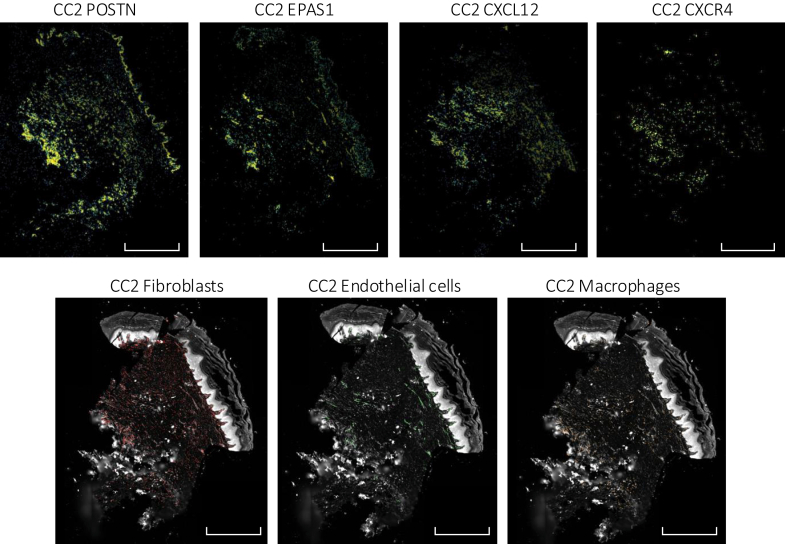
**Spatial representation of genes and cell types in CC2.** Top row: spatial expression maps of key differentially expressed genes in CC2, including POSTN (periostin), EPAS1 (HIF-2α), CXCL12, and CXCR4, visualized using the Xenium In Situ platform. Bottom row: spatial distribution of key cell populations in CC2, including fibroblasts (red), endothelial cells (green), and macrophages (orange). Bar = 1 mm. CC, calcinosis cutis; HIF-2α, hypoxia-inducible factor 2α.

To spatially contextualize the transcriptional findings identified in the differential gene expression analysis, we visualized the distribution of key upregulated genes and major cell populations across both CC tissue sections using the Xenium In Situ platform. In both samples, *POSTN* expression was concentrated along the interface and within the dermal compartment surrounding the calcinosis deposit. *EPAS1* and *CXCL12* also demonstrated broad regional enrichment across the viable tissue, whereas *CXCR4* showed a sparser, punctate distribution pattern that correlated with macrophage localization (Figures 9 and 10).

## Discussion

### Key findings and overview

To our knowledge, spatial transcriptomic analysis of CC in CCLE has not been previously reported. A few key findings within the study were identified. First, fibroblasts within CC lesions demonstrate significant transcriptional upregulation of osteogenic and ECM-related genes. Second, endothelial cells in these lesions show activation of hypoxic response pathways. Third, specific immune cell populations, particularly DCs and macrophages, exhibit distinct activation signatures, establishing potential chemotactic axes that recruit these cells to sites of calcification. Finally, these pathological changes demonstrate clear spatial organization, with specific cellular and molecular alterations concentrated in pericalcinosis regions. This spatial specificity suggests that CC is driven by localized pathogenic niches that collectively promote mineralization.

### Fibroblast-mediated mineralization

In fibroblasts of CC-associated lesions, several key genes, including *POSTN*, *SFRP2*, and various collagen-related and metalloproteinase genes (*COL6A* family, *MMP2*), were upregulated, suggesting ECM restructuring and pro-osteogenic cellular patterns. *POSTN,* which encodes periostin, a matricellular protein natively involved in collagen fibrillogenesis and tissue repair (Norris et al, 2007), has been shown to influence tissue stiffness in fibrotic diseases, where it is induced by inflammatory cytokines to directly promote arterial calcification (Lin et al, 2016; Schwanekamp et al, 2016). *SFRP2* (secreted frizzled related protein 2), a modulator of Wnt signaling, has also been implicated in cardiac fibrocalcification through upstream activation of tissue-nonspecific alkaline phosphatase. In CLE specifically, SFRP2+ fibroblast subsets exhibit increased inflammatory profiles and altered responses to TGF-β, contributing to profibrotic outcomes (Shoffner-Beck et al, 2024; Tabib et al, 2018).

These findings align with cellular trends observed in other calcifying disorders. In dermatomyositis-associated calcinosis, spatial transcriptomic profiling similarly identified the upregulation of matrix metalloproteinases, including *MMP1*, *MMP9*, and *MMP13*, as well as cartilage- and bone-specific collagens, suggesting conserved ECM remodeling mechanisms across calcifying autoimmune skin diseases (Parks et al, 2025). Pathological heart fibroblasts have been shown to adopt osteogenic fates, directly contributing to heart muscle mineralization (Li et al., 2021; Rutkovskiy et al, 2017). Similarly, in atherosclerosis and vascular calcification, vascular smooth muscle cells and neighboring fibroblasts undergo persistent disorganized ECM secretion, with senescent vascular smooth muscle cells and fibroblasts notably overexpressing collagen and genes associated with bone calcification (Mas-Bargues et al, 2022; Pustlauk et al, 2020). In systemic sclerosis, dysregulated bone metabolism, local phosphate disturbances (potentially from acro-osteolysis or apoptotic cells), and an imbalance between mineralization promoters and inhibitors promote dermal fibroblast osteoblastic differentiation (Burgess et al, 2021; Valenzuela and Chung, 2022). This is supported by the successful transdifferentiation of human dermal fibroblasts into osteoblast-like cells under specific conditions and evidence suggesting that systemic sclerosis fibroblasts adopt a pro-osteogenic profile (Pihlström et al, 2022). The shared deposition of hydroxyapatite crystals in both vascular and cutaneous calcification further supports these mechanistic similarities, because ectopic calcification often resembles normal bone mineralization processes (Le and Bedocs, 2023). The commonality of these mechanisms across diverse conditions, also seen in chronic kidney disease mineral bone disorder or rare genetic calcification syndromes, underscores that ectopic calcification is an active, cell-mediated process involving fibroblast dysregulation (Thompson and Towler, 2012).

### Vascular dysfunction and hypoxia

CC-associated endothelial cells demonstrated enrichment of hypoxic response pathways and consistent upregulation of *EPAS1* (endothelial PAS domain protein 1)/hypoxia-inducible factor (HIF)-2α, a key component of the HIF complex, which plays a critical role in the body's adaptation to changing oxygen levels (Negri, 2022; Semenza, 2014). Whereas HIF-1α is often associated with acute hypoxic responses, HIF-2α is predominantly expressed in the vascular endothelium and is more involved in responses to chronic or mild hypoxia, triggering angiogenesis and erythropoiesis (Lee et al, 2020; Manalo et al, 2005).

In the context of lupus, endothelial dysfunction represents a well-established systemic complication that bridges the gap between local cutaneous manifestations and systemic vascular pathology. IFN-I, a central mediator in systemic lupus erythematosus pathogenesis, directly induce endothelial destabilization and dysfunction, with excessive IFN-I causing depletion of endothelial progenitor cells and being linked to increased cardiovascular risk in systemic lupus erythematosus (Moschetti et al, 2022; Thacker et al, 2010). This IFN-I–mediated endothelial dysfunction has been shown to be amplified by neutrophil extracellular traps through NETosis, which directly damages endothelial cells and activates matrix metalloproteinases (Garcia-Romo et al, 2011; Salemme et al, 2019).

Hypoxia's role in pathological calcification is also well-established in other contexts. In atherosclerosis, hypoxia can directly induce osteogenic differentiation and endothelial–mesenchymal transition pathways in vascular tissue, promoting nucleation sites for calcification (Aikawa and Blaser, 2021; Bakhshian et al, 2017; Balogh et al, 2019). A hypoxic microenvironment is also a critical factor in the pathogenesis of autoimmune skin diseases (Gong et al, 2024; McGettrick and O’Neill, 2020). For systemic sclerosis, vascular dysfunction and hypoxia are mechanistically linked to calcinosis through ischemia–reperfusion injury and mechanical stress (Davuluri et al, 2024). Vascular dysfunction and hypoxia are also implicated in juvenile dermatomyositis–associated calcinosis, with studies suggesting a specific role for ROS and mitochondrial dysfunction in muscle cells (Davuluri et al, 2022; Preuße et al, 2016).

The current findings suggest that similar hypoxia-driven mechanisms may occur in CLE, where tissue damage and local infiltration of immune cells may impair local perfusion, depriving it of localized oxygen. The upregulation of HIF-2α rather than the acute-phase HIF-1α further suggests that these processes likely occur over an extended duration.

### Immune cell contributions

Macrophage populations in CC lesions exhibited differential regulation of several key genes, including caspases (*CASP1* and *CASP4*). These caspases are recognized for their involvement in inflammasome activation and pyroptosis, an inflammatory form of programmed cell death (Anderson et al, 2023; Fernández-Duran et al, 2022). Importantly, caspase-1 is among the IFN-stimulated genes that are upregulated in lupus; recent studies demonstrate that prolonged type I IFN exposure, as seen in patients with systemic lupus erythematosus, primes monocytes for robust inflammasome activation in an IRF-1–dependent manner (Liu et al, 2017). The activation of inflammasomes, particularly the NLRP3 inflammasome, has been identified as a response to calcification, with type I IFN–mediated upregulation of NLRP3 creating a mechanistic link between the characteristic IFN signature of lupus and calcium-induced inflammatory responses (Chen and Li, 2024; Yu et al, 2021). This suggests a potential interplay where existing calcium deposition may further contribute to inflammatory responses, creating a positive feedback loop.

Macrophage populations in CC lesions also demonstrated elevated *CXCR4* expression, with a corresponding upregulation of its ligand, *CXCL12*, in endothelial cells, representing a chemotactic axis for directed macrophage recruitment to calcification sites. Spatial neighborhood analysis corroborates this chemotactic axis at the tissue level, with macrophages and endothelial cells showing reciprocal spatial proximity. Pathway analysis further revealed enrichment for inflammatory response regulation, chemotaxis, and myeloid cell activation in macrophages. The CXCL12–CXCR4 axis is a well-characterized signaling pathway critical to immune cell recruitment, cell migration, and tissue repair in both cardiovascular and autoimmune diseases (Döring et al, 2014; García-Cuesta et al, 2019). Specifically, *CXCL12* binding to *CXCR4* mediates the homing and retention of progenitor cells and inflammatory cells at sites of injury or inflammation. In lupus calcinosis, this axis may facilitate the robust migration and retention of macrophages to pericalcinosis regions, where they contribute to the local inflammatory environment.

Beyond recruitment, macrophages have established roles in atherosclerotic calcification, where proinflammatory macrophages may release various cytokine mediators that reportedly promote the osteogenic differentiation of vascular cells and contribute to ECM remodeling (Hansson and Libby, 2006; Tylutka et al, 2024). Macrophages are also recognized as being sensitive to hypoxia, which can polarize them toward a proinflammatory (M1-like) phenotype that exacerbates tissue damage and calcification. Hypoxic macrophages have also been observed to release procalcific extracellular vesicles, which may contribute to nucleation sites for calcium deposition (Qi et al, 2025; Yakupova et al, 2022).

Our study also noted nonspecific enrichment of lymphocyte differentiation and inflammatory response regulation in DCs of CC lesions. Although the specific role of DCs in CC pathogenesis was not fully elucidated in this study, DCs are central to lupus pathogenesis as major producers of type I IFNs. Both plasmacytoid DCs and conventional DCs are implicated in producing type I IFN and driving the IFN signature characteristic of lupus (Klarquist et al, 2016; Postal et al, 2020). Hypoxia can influence DC maturation and function, enhancing their proinflammatory roles and contributing to a sustained hypoxic environment (Jantsch et al, 2008; Tran et al, 2020).

### Limited keratinocyte and T-cell involvement

Keratinocyte and T-cell populations showed minimal transcriptional differences between the CC and noCC groups, suggesting that these cells may play less direct roles in the mineralization process itself, despite their importance in broader CLE pathogenesis. This transcriptional finding was reinforced by spatial neighborhood analysis, in which 87% of keratinocyte neighbors were other keratinocytes, reflecting their epidermal compartmentalization and physical separation from the pericalcinosis stromal and immune cell populations.

### Pericalcinosis region specificity

Spatial transcriptomics revealed distinct region-specific expression patterns within CC samples, indicating that calcification in CCLE may represent a more localized rather than diffuse pathological process. Within their respective cell types, notable genes (eg, *POSTN*, *EPAS1*, *SFRP2*, *CXCL12*, *CXCR4*) were not only globally upregulated but also enriched within pericalcinosis regions. This recapitulation of themes at the local level further supports the idea that there may be microenvironment “hotspots” required for initiating and sustaining mineralization.

### Generating the procalcific environment

Spatial proximity analysis revealed reduced intercellular distances between CXCL12+ endothelial cells and CXCR4+ macrophages as well as between EPAS1+ endothelial cells and both CXCR4+ macrophages and POSTN+ fibroblasts in CC samples. These findings suggest localized and enhanced cell–cell communication within pericalcinosis niches. These spatial relationships point toward a collective contribution of various cellular processes to the pathogenesis of CC.

We hypothesize that chemokine signaling, such as through the CXCL12–CXCR4 axis, plays a pivotal role in facilitating robust macrophage recruitment and retention at calcification sites. This recruitment may influence the local inflammatory milieu classically described in lupus, such as TNFα, IFN-1, or neutrophil extracellular trap–related signaling, and drive tissue remodeling, particularly in endothelial cells and fibroblasts, within these niches. This inflammatory damage may trigger and potentiate hypoxia, mediated by factors such as EPAS1. Alongside an enhancement of the proinflammatory and procalcific functions of macrophages, these factors could promote osteogenic differentiation in nearby fibroblasts and endothelial cells. Collectively, these increased osteogenic processes, including ECM dysregulation, create a critical scaffold for mineralization. This observed spatial organization represents a functional arrangement that could reinforce a calcification process. The resulting tissue damage and calcium deposition not only impair vascular function and exacerbate hypoxia, thereby perpetuating the cycle, but may also drive secondary immune cascades through caspase-mediated inflammasome pathways, further amplifying the procalcific environment.

### Limitations

This study has several important limitations. The small sample size of 4 patients (2 CC, 2 noCC) limits generalizability and statistical power to detect subtle differences or rare cell populations. Heterogeneous treatment status at biopsy may also introduce potential confounding factors because systemic treatment may influence gene expression profiles independently of disease processes.

Another limitation is the restricted gene panel, comprising 260 genes from the Human Skin Panel supplemented with 100 custom genes, totaling 360 targeted transcripts. Although this panel was curated to capture major cell types and disease-relevant pathways in cutaneous lupus, the targeted nature of this approach precluded comprehensive subpopulation analysis within the identified cell clusters. Future studies employing whole-transcriptome spatial approaches, such as Visium HD or Xenium, with expanded custom panels would enable higher-resolution cell subtype mapping and more granular characterization of the activated cellular states within pericalcinosis niches.

In addition, transcriptomic data provide correlational rather than causal insights. Although this study identified activated pathways and altered cell–cell interactions, functional validation is required to establish causality. More detailed histological characterization of CLE pathology in CC samples and specific calcinosis locations within the dermis could provide important contextual information about the inflammatory state surrounding calcification.

Validation in larger, independent cohorts that include diverse CCLE forms and calcinosis presentations is essential to enhance generalizability. Functional studies, including in vitro fibroblast differentiation assays, coculture models, and in vivo animal models, are needed to confirm causal roles of identified genes and pathways. Investigation of specific inflammatory triggers that initiate procalcifying programs in fibroblasts and endothelial cells should be pursued through analysis of patient serum or tissue fluid and targeted in vitro stimulation studies.

In summary, this spatial transcriptomics study offers, to our knowledge, previously unreported insights into the cellular and molecular landscape of CC in CCLE. The findings demonstrate fibroblast transcriptional reprogramming toward osteogenic phenotypes, endothelial hypoxic responses, and immune cell activation signatures within spatially organized pericalcinosis regions. These mechanisms resemble those seen in atherosclerosis and calcinosis in other autoimmune conditions, indicating conserved pathways of ectopic calcification across different diseases. Although limited by sample size, these results identify potential therapeutic targets and lay the groundwork for future mechanistic and translational studies to address this challenging complication in patients with lupus.

## Materials and Methods

### Patient selection and clinical characterization

Patients with CCLE were identified on the basis of clinical and histopathological criteria. The inclusion criteria included (i) a clinical diagnosis of CCLE confirmed by dermatopathology and (ii) age ≥18 years. Patients were stratified into 2 groups, those with CC and those without CC, on the basis of histopathological assessment. The clinical data collected included demographics, clinical characteristics, and current medications.

### Tissue collection and processing

Skin biopsies were obtained from lesional areas using 4-mm punch biopsies under local anesthesia with 1% lidocaine. For patients with CC, biopsies were taken from areas with visible or palpable calcification. For control patients without CC, biopsies were obtained from representative CLE lesions. Tissue samples were immediately placed in formalin and snap frozen in liquid nitrogen. Samples were stored at −80 °C until processing.

### Xenium assay

Spatial transcriptomic analysis was performed using the Xenium In Situ platform (10x Genomics, Pleasanton, CA) with the Human Skin Panel, which targets 260 genes relevant to tissue architecture, immune function, and disease pathways, along with an additional 100 custom genes related to previously implicated inflammatory processes in cutaneous disease (Table 6). The assay was performed according to the manufacturer’s instructions, including in situ hybridization, signal amplification, and imaging cycles. Posthybridization processing involved DAPI staining for nuclear visualization.

**Table 6:** Custom Panel of Genes Implicated in Inflammatory Skin Conditions

Gene Names
ADAM10, ADAM17, ADAR, AHR, AIM2, AIP, ARNT, ATG14, BAG1, BAX, BBC3, BCL2, BECN1, BID, BIK, BIRC2, BMF, CARD16, CARD18, CARD8, CASP1, CASP10, CASP14, CASP4, CASP8, CD40, CEBPA, CHUK, CISD2, DLL1, DLL4, EDA2R, EIF4EBP1, EPAS1, EPHA3, FADD, FAS, GLIPR2, GSDMA, GSDMB, GSDMC, GSDMD, HOXA9, IL1R2, ITCH, JAG1, LAMP1, MAF, MALT1, MAML1, MEFV, MLST8, MME, MSRB2, MTOR, NEDD8, NLRP1, NLRP10, NLRP12, NLRP3, NOD2, NOTCH1, NOTCH2, PIDD1, PIK3C3, PIK3R4, PRKAA1, PRKAA2, PYCARD, RAB39B, RAB6A, RB1, RBPJ, RFFL, RIPK1, RIPK2, RIPK3, RPTOR, SCAP, SEL1L, SMAD2, SMPD3, TAX1BP1, TICAM1, TICAM2, TLR3, TNF, TNFAIP3, TNFSF9, TNIP1, TRADD, TRAF2, TRAF3, TRIM38, TSPAN14, TSPAN15, TSPAN5, UVRAG, ZBP1, ZFAND5

### Image acquisition

High-resolution fluorescence images were acquired using the Xenium Analyzer with automated image acquisition protocols. Images were captured at multiple z-planes to ensure the complete capture of transcriptomic signals throughout the tissue section.

### Data processing and quality control

Raw Xenium data were processed using the Xenium Onboard Analysis pipeline (10x Genomics) to generate gene expression matrices and spatial coordinates for each detected transcript. Quality control metrics included assessments of transcript detection efficiency, background signal levels, and spatial distribution patterns.

Data analysis was performed in R (version 4.3.0) using the Seurat package (version 5.0), which was specifically designed for spatial transcriptomics analysis (Hao et al, 2021). Quality control filters were applied to remove low-quality cells on the basis of the following criteria: (i) a minimum of 10 detected genes per cell, (ii) a maximum of 5000 detected genes per cell to exclude potential multiplets, and (iii) cells with spatial coordinates outside the tissue boundary.

### Sample integration and normalization

Individual sample datasets were merged into a unified Seurat object for comparative analysis (Butler et al, 2018; Stuart et al, 2019). Data normalization was performed using the SCTransform method to account for technical variation and differences in transcript capture efficiency between samples (Hafemeister and Satija, 2019). Highly variable genes were identified using the vst method, and principal component analysis was performed.

### Clustering and cell-type identification

Unsupervised clustering was performed using the Leiden algorithm implemented in Seurat (Traag et al, 2019). The optimal cluster resolution was determined by evaluating cluster stability and biological interpretability across multiple resolution parameters (0.1–1.0).

Cell-type identification was accomplished through a 2-step approach. Initial cell-type assignments were made using the BluePrint and Encode reference databases through the SingleR package, which provides curated gene expression profiles for major cell types (ENCODE Project Consortium, 2012; Martens and Stunnenberg, 2013). Automated classifications were refined through manual inspection of canonical marker gene expression patterns (Table 7).

**Table 7:** Canonical Markers Used to Aid in Cluster Identification after SingleR Labeling

Cell Types	Markers
Fibroblasts	COL1A1, LUM, PDGFRA
Keratinocytes	KRT5, KRT15, TP63
Macrophages	CD68, C1QA, LYZ
T cells	CD3E, CD3D, CD3G
Dendritic cells	CLEC9A, CLEC10A, CD1A
Endothelial cells	CDH5, CLDN5

### Differential expression analysis

Differential gene expression analysis was performed to identify genes specifically associated with CC. Comparisons were made between (i) CC and noCC samples within different cell types and (ii) pericalcinosis regions and noncalcinosis regions within CC samples. Statistical testing was performed using the Wilcoxon rank-sum test implemented in Seurat, with Bonferroni correction for multiple testing. Genes with a FDR < 0.05 and an absolute log_2_ FC > 0.25 were considered significantly differentially expressed.

### Pathway analysis

Gene ontology enrichment analysis was performed using the clusterProfiler package to identify biological pathways associated with differentially expressed genes (Yu et al, 2012). Pathway enrichment was assessed using hypergeometric testing with Benjamini–Hochberg correction for multiple testing (adjusted *P* < .05).

### Spatial visualization

All spatial analyses and cell-type distributions were visualized using a custom R script and the Seurat or ggplot2 packages. Spatial plots were generated to show the distribution of cell types, gene expression levels, and differential expression patterns across tissue sections.

### Spatial neighborhood composition analysis

Nearest-neighbor identification was performed using the RANN package (version 2.6.1) in R, implementing the nn2 function with a fixed radius of 50 μm and a maximum of k = 30 neighbors per cell. For each cell, all neighboring cells within the defined radius were identified, and their cell-type annotations were recorded. Neighbor proportions were calculated by dividing the count of each neighboring cell type by the total number of neighbors for each cell, with the results aggregated by cell type. The resulting neighborhood composition matrix was visualized as a heatmap using the pheatmap package, with hierarchical clustering applied to both rows and columns. The analysis was performed on CC samples only, given the focus on characterizing the pericalcinosis cellular niche.

## Ethics Statement

This study was conducted in accordance with the Declaration of Helsinki and approved by the Johns Hopkins Institutional Review Board (IRB00356520). For retrospectively identified patients, whose biopsies were obtained for clinical care, consent was waived by the institutional review board, and clinical data were reviewed retrospectively. Written informed consent was obtained from patients who were prospectively enrolled for tissue collection.

## Data Availability Statement

Datasets related to this article can be found at https://data.mendeley.com/datasets/zcf4fy9j65/1, an open-source online data repository hosted on Mendeley Data.

## ORCIDs

Aaron Bao: http://orcid.org/0000-0002-2069-9985

Saloni Patel: http://orcid.org/0000-0002-8250-9569

Sewon Kang: http://orcid.org/0000-0002-7841-9392

Jaroslaw Jedrych: http://orcid.org/0000-0003-2385-7085

Martin Alphonse: http://orcid.org/0000-0003-3447-1284

Jun Kang: http://orcid.org/0009-0001-7883-0798

## Conflicts of Interest

The authors state no conflict of interest.
